# Female Authors in Nuclear Medicine Journals: A Survey from 2014 to 2020

**DOI:** 10.2967/jnumed.121.262773

**Published:** 2022-07

**Authors:** Charline Lasnon, Gilles Girault, Rachida Lebtahi, Catherine Ansquer, Justine Lequesne, Elske Quak

**Affiliations:** 1 Nuclear Medicine Department, Comprehensive Cancer Center F. Baclesse, Unicancer, Caen, France;; 2 Normandy University, UNICAEN, INSERM 1086 ANTICIPE, Caen, France;; 3 Medical Library, Comprehensive Cancer Center F. Baclesse, Unicancer, Caen, France;; 4 Department of Nuclear Medicine, Beaujon Hospital, Clichy, France;; 5 Department of Nuclear Medicine, University Hospital of Nantes, Nantes, France;; 6 University of Nantes and CNRS, INSERM, CRCINA, Nantes, France; and; 7 Biostatistics Department, Comprehensive Cancer Center F. Baclesse, Unicancer, Caen, France

**Keywords:** physicians, women, authorship, nuclear medicine

## Abstract

Despite the feminization of the medical workforce, women do not have the same career perspectives as men. In nuclear medicine, little information is available on the sex gap regarding prominent author positions in scientific articles. Therefore, the purpose of this study was to evaluate recent trends in the sex distribution of first and last authorship of articles published in nuclear medicine journals. **Methods:** We conducted a bibliometric analysis of first and last author sex of articles published from 2014 to 2020 in 15 nuclear medicine journals. Manuscript title, article type, journal impact factor, date of publication, and first and last name and country of provenance of first and last authors were noted. The Gender API software was used to determine author sex. All statistics were descriptive. **Results:** Women represented 32.8% of first authors and 19.6% of last authors. Female authorship increased from 28.2% (428 of 1,518 articles) in 2014 to 35.5% (735 of 2,069 articles; relative increase, 72%) in 2020 (*P* < 0.001) for first authors and from 15.6% (237 of 1,518 articles) in 2014 to 20.5% (424 of 2,069 articles; relative increase, 79%) in 2020 (*P* < 0.001) for last authors. Parity was forecast for 2035 for first authors and 2052 for last authors. Female authorship increased in Europe for first authors (*P* = 0.014) and last authors (*P* < 0.001), in high-ranking journals for first authors (*P* = 0.004) and last authors (*P* < 0.001), and in other journal ranks for last authors (*P* = 0.01). Female first and last authorship rose for original articles (*P* = 0.02 and *P* = 0.01, respectively) and case reports (*P* < 0.001 and *P* = 0.002, respectively). Regarding collaborations, the proportion of articles produced by male first and last authors decreased from 62.2% in 2014 to 52.9% in 2020 in favor of female first and last authors (odds ratio, 1.07; *P* < 0.001), male first and female last authors (odds ratio, 1.05; *P* < 0.001), and female first and male last authors (odds ratio, 1.03; *P* < 0.001). **Conclusion:** Female first and last authorship in nuclear medicine journals increased substantially from 2014 to 2020, in particular in high-ranking journals, in Europe, and for original articles and case reports. Male-to-male collaborations decreased by 10% in favor of all other collaborations. Parity can be foreseen in a few decades.

## Visual Abstract

**Figure:**
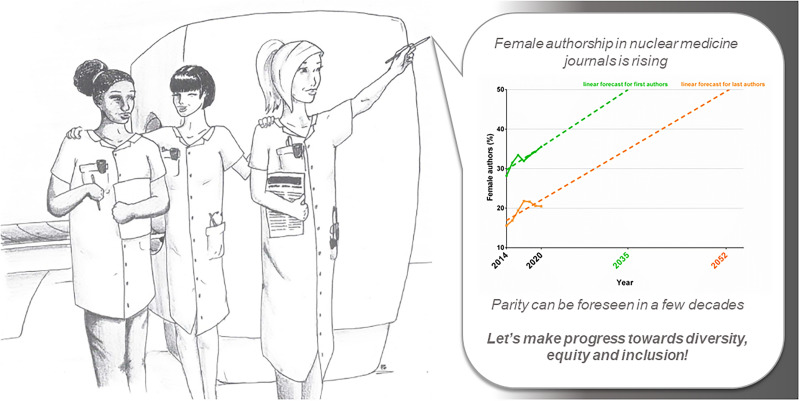

Despite efforts to offset the tendency, sex gaps and prejudices broadly persist in modern-day society, and despite the feminization of the workforce in medicine, women do not have the same career perspectives or pay as men (1–4). In the field of medical imaging, the pipeline to the top positions has been described as leaky for female talent, and leadership positions are held predominantly by men (5–10).

Regarding nuclear medicine, the literature on the sex gap and sex-related career challenges is scarce. A recent study reported the underrepresentation of women in academic and leadership positions compared with men in North America and Canada, despite equal academic performance (11). In Europe, a 2007 membership survey of the European Association of Nuclear Medicine showed that one third of physicians were women, with an increasing percentage of female physicians over time and at a younger age (12). However, the sex distribution varied widely between countries, and the evolution of the sex gap in nuclear medicine over time and higher on the career ladder has received little attention.

As scientific publishing is a key factor for career advancement, trends in the sex distribution of prominent author positions may reflect future evolution of women toward leadership positions. Therefore, the main objective of the current study was—through a descriptive bibliometric analysis—to evaluate recent trends in the sex distribution of the most prestigious author positions, that is, first and last authorship, in articles published in anglophone nuclear medicine journals from 2014 to 2020.

## MATERIALS AND METHODS

This study was exempt from local institutional review board approval.

We performed a PubMed search for 2014 to 2020 to retrieve all articles published in the 15 purely anglophone nuclear medicine journals in the “Radiology, Nuclear Medicine, and Medical Imaging” category of the Journal Citation Reports 2019: *Journal of Nuclear Medicine, European Journal of Nuclear Medicine and Molecular Imaging, Clinical Nuclear Medicine, Seminars in Nuclear Medicine, Journal of Nuclear Cardiology, Molecular Imaging and Biology, Molecular Imaging, EJNMMI Research, Annals of Nuclear Medicine, EJNMMI Physics, Nuclear Medicine and Biology, Contrast Media & Molecular Imaging, Quarterly Journal of Nuclear Medicine and Molecular Imaging, Nuclear Medicine Communications,* and *Hellenic Journal of Nuclear Medicine.* The bibliographic references of all articles were imported into the bibliographic data management software Endnote. An import filter was created to add the following PubMed bibliographic data to the usual bibliographic fields: publication date, first and last name of all authors, affiliation addresses, and article type. This dataset was exported to Excel, and the following variables were recorded for each entry: manuscript title, publication year, first and last name of the first and last authors, article type, journal impact factor according to the Journal Citation Reports 2019, and country of provenance of first and last authors. The Gender API software (https://gender-api.com/) was used to determine the sex of the first and last authors. Performance metrics of this software can be found elsewhere (13). The date of censoring for 2020 was February 24, 2021. Preprints of 2020 were excluded. In the event of missing data, entries were excluded, as were entries with a single author. The following article types were excluded: “Published Erratum,” “Retracted Publication,” “News,” “Lecture,” “Historical Article,” “Biography,” “Portrait,” “Introductory Journal Article,” and “English Abstract.”

The main aim of the study was to analyze the evolution of the percentages of female first and last authorship over the study period. Secondary aims were to forecast the year in which parity will be attained for first and last authors; to evaluate sex distributions according to continent, journal rank, and article type; and to evaluate collaborations between the sexes. For the analysis of author sex according to provenance, countries were classified according to continent. For the analysis of author sex according to journal rank, references were classed as high-ranking (journal impact factor, 7.887–6.622) or others (journal impact factor, 3.544–0.982). For the analysis of author sex according to article type, references were categorized as original article, review, case report, and editorial/letter. References tagged solely as “Journal Article” by PubMed were categorized as original article.

Collaboration between first and last author sex was explored by classifying articles in the 4 following categories: male first and last authors, female first and male last authors, male first and female last authors, and female first and last authors.

All statistics were descriptive. Fisher exact tests were used to analyze the distribution of female authorship from 2014 to 2020. Linear regression was used to forecast the year in which parity for first and last authorship will be reached. A multinomial logistic regression model was constructed to measure the evolution of the distribution of collaborations over time, in which male first and male last authorship was considered the reference. Graphic and statistical analyses were performed on XLSTAT Software (XLSTAT 2007: Data Analysis and Statistical Solutions for Microsoft Excel; Addinsoft, 2017) and R Software (version 4.0.2). For all statistical tests, a 2-tailed *P* value of less than 0.05 was considered statistically significant.

## RESULTS

### Data Characteristics

In total, 15,720 references were imported, of which 12,450 (79.2%) fulfilled the article type criteria and presented complete data regarding first and last author sex and provenance. Data characteristics are presented in Table 1.

**TABLE 1.** Data Characteristics

Variable	Data
Number of publications	12,450
Year	
2014	1,518 (12.2)
2015	1,594 (12.8)
2016	1,783 (14.3)
2017	1,869 (15)
2018	1,660 (13.3)
2019	1,957 (15.7)
2020	2,069 (16.6)
First-author sex	
Female	4,082 (32.8)
Male	8,368 (67.2)
First-author continent	
Africa	64 (0.5)
Asia	3,370 (27.1)
Europe	5,699 (45.8)
North America	2,985 (24.0)
Oceania	211 (1.7)
South America	121 (1.0)
Last-author sex	
Female	2,445 (19.6)
Male	10,005 (80.4)
Last-author continent	
Africa	62 (0.5)
Asia	3,290 (26.4)
Europe	5,638 (45.3)
North America	3,135 (25.2)
Oceania	217 (1.7)
South America	108 (0.9)
Journal rank*	
High-ranking	6,205 (49.8)
Others	6,245 (50.2)
Article type	
Original article	8,612 (69.2)
Review	1,017 (8.2)
Case report	2,394 (19.2)
Editorials/letters	427 (3.4)

* High-ranking = impact factor of 7.887–6.622; others = impact factor of 3.544–0.982.

Data are number followed by percentage.

### First Authors

**FIGURE 1.**
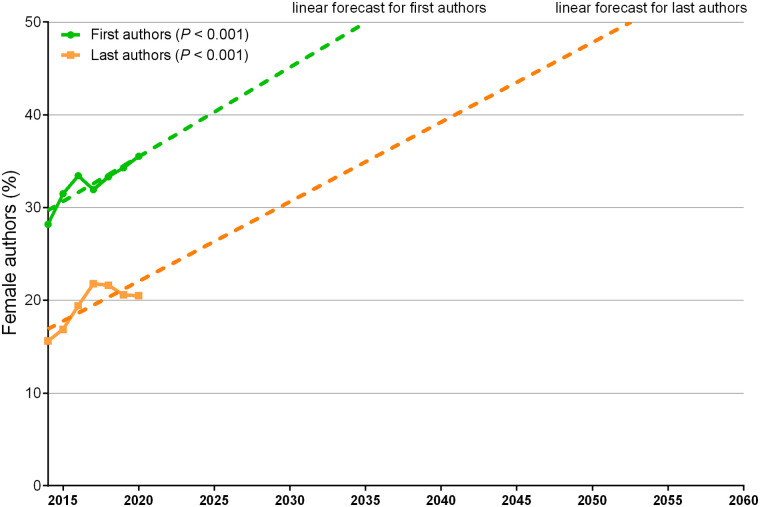
Female authorship increased from 28.2% in 2014 to 35.5% in 2020 (*P* < 0.001) for first authors and from 15.6% in 2014 to 20.5% in 2020 (*P* < 0.001) for last authors. For female last authors, peak of 21.8% was observed in 2017. Linear forecasts show that at current rate, parity is predicted in 2035 for first authors and in 2052 for last authors.

**FIGURE 2.**
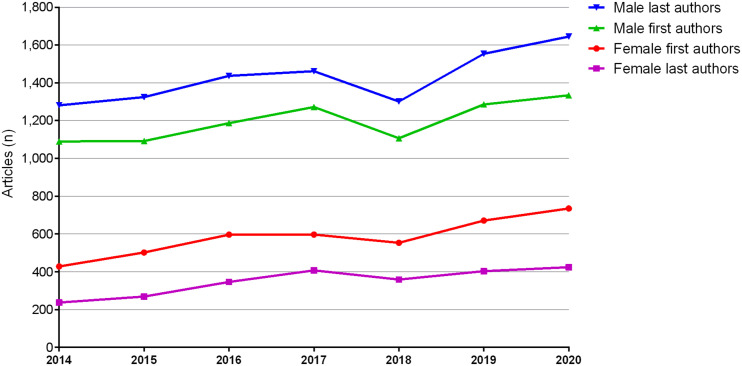
Absolute numbers of articles for female and male first and last authors from 2014 to 2020. Articles by female first authors increased from 428 of 1,518 in 2014 to 735 of 2,069 in 2020, whereas articles by male first authors increased from 1,090 of 1,518 in 2014 to 1,334 of 2,069 in 2020. Articles by female last authors increased from 237 of 1,518 in 2014 to 424 of 2,069 in 2020, whereas articles by male last authors increased from 1,281 of 1,518 in 2014 to 1,645 of 2,069 in 2020.

Overall, 4,082 of 12,450 (32.8%) first authors were female (Table 1). Female first authorship increased over time from 428 of 1,518 (28.2%) in 2014 to 735 of 2,069 (35.5%) in 2020 (*P* < 0.001) (Fig. 1), representing a relative increase of 72% in 7 y (+307 articles). At this rate, parity was forecast for 2035 (Fig. 1). Conversely, male first authorship increased by 22% between 2014 and 2020 (+244 articles). Detailed absolute numbers of articles for each year between 2014 and 2020 for female and male first authors are depicted in Figure 2.

**FIGURE 3.**
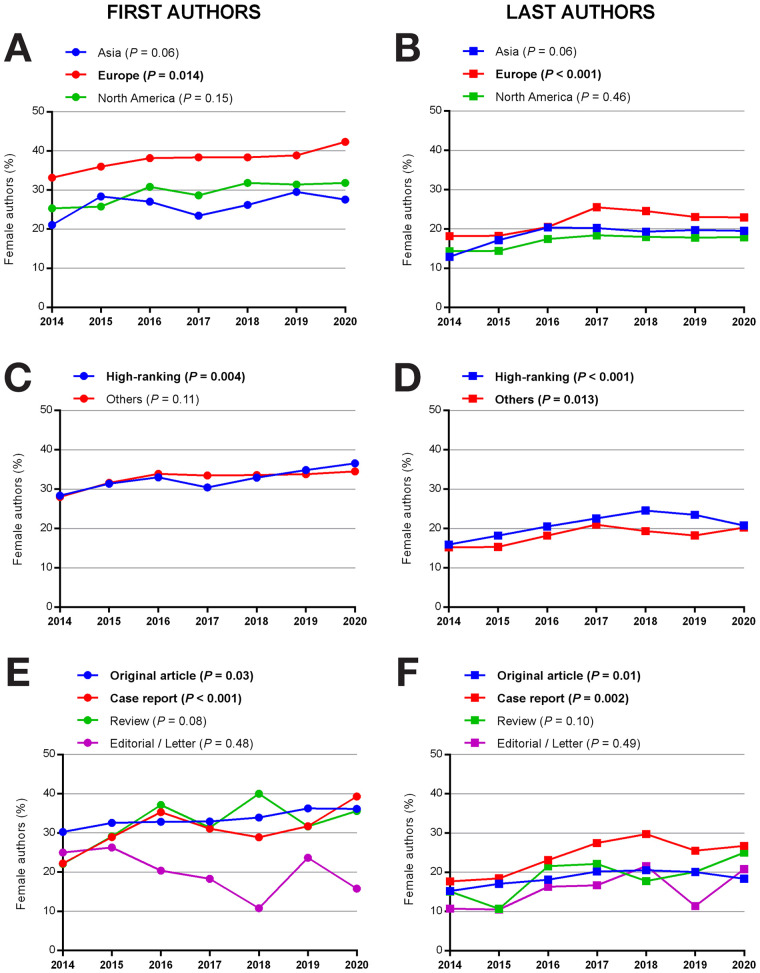
Percentage of female first authorship from 2014 to 2020 according to continent of provenance (A), journal rank (C), and article type (E), and percentage of female last authorship from 2014 to 2020 according to continent of provenance (B), journal rank (D), and article type (F). Bold values are statistically significant. Journals were ranked according to impact factor: high-ranking (impact factor, 7.887–6.622) or others (impact factor, 3.544–0.982).

Regarding the geographic provenance of first authors, 12,054 of 12,450 (96.8%) articles came from 3 continents: Asia (3,370 of 12,450 [27.1%]), Europe (5,699 of 12,450 [45.8%]), and North America (2,985 of 12,450 [24.0%]). Data from Africa, Oceania, and South America were insufficient to be included in the analysis and can be found in Supplemental Figure 1 (supplemental materials are available at http://jnm.snmjournals.org). In Europe, female first authorship increased from 232 of 700 articles (33.1%) in 2014 to 385 of 910 articles (42.3%) in 2020 (*P* = 0.014). In Asia and North America, percentages of female first authorship per year did not significantly differ from 2014 to 2020 (*P* = 0.06 and *P* = 0.15, respectively) (Fig. 3A).

Regarding journal rank, percentages of female first authorship in high-ranking journals increased from 240 of 847 articles (28.3%) in 2014 to 371 of 1,015 articles (36.6%) in 2020 (*P* = 0.004). No changes were observed for the other journal ranks (*P* = 0.11) (Fig. 3C).

Regarding article type, female first authorship increased for original articles from 338 of 1,116 articles (30.3%) in 2014 to 516 of 1,428 articles (36.1%) in 2020 (*P* = 0.03) and for case reports from 64 of 288 articles (22.2%) in 2014 to 153 of 389 articles (39.3%) in 2020 (*P* < 0.001). No change was observed for reviews or editorials/letters over time (*P* = 0.08 and 0.48, respectively). Female first authors were underrepresented in the category editorial/letters (Fig. 3E).

### Last Authors

Overall, 2,445 of 12,450 (19.6%) last authors were female (Table 1). Female last authorship increased over time from 237 of 1,518 (15.6%) in 2014 to 424 of 2,069 (20.5%) in 2020, representing a relative increase of 79% (+187 articles), with a peak of 21.8% in 2017 (*P* < 0.001) (Fig. 1). Parity was forecast for 2052 (Fig. 1). Conversely, male first authorship increased by 28% between 2014 and 2020 (+364 articles). Detailed absolute numbers of articles for each year between 2014 and 2020 for female and male last authors are depicted in Figure 2.

Regarding the geographic provenance of last authors, 12,063 of 12,450 (96.9%) articles again came from 3 continents: Asia (3,290 of 12,450 [26.4%]), Europe (5,638 of 12,450 [45.3%]), and North America (3,135 of 12,450 [25.2%]). Data from Africa, Oceania, and South America can be found in Supplemental Figure 1. In Europe, female last authorship increased from 126 of 693 articles (18.2%) in 2014 to 208 of 906 articles (23.0%) in 2020, with a peak of 25.5% in 2017 (*P* < 0.001). In Asia and North America, percentages of female last authorship per year did not significantly differ from 2014 to 2020 (*P* = 0.06 and *P* = 0.46, respectively) (Fig. 3B).

Regarding journal rank, female last authorship increased in high-ranking journals from 135 of 847 articles (15.9%) in 2014 to 211 of 1,015 articles (20.8%) in 2020, with a peak of 24.6% in 2018 (*P* < 0.001) and in other-ranking journals from 102 of 671 articles (15.2%) in 2014 to 213 of 1,054 (20.2%) in 2020 (*P* = 0.013) (Fig. 3D).

Regarding article type, female last authorship increased from 170 of 1,116 articles (15.2%) in 2014 to 262 of 1,428 articles (18.3%) in 2020 (*P* = 0.01) for original articles and from 51 of 288 articles (17.7%) in 2014 to 104 of 389 articles (26.7%) in 2020 for case reports, with a peak of 29.7% in 2018 (*P* = 0.002). No change was observed for reviews or editorials/letters (*P* = 0.10 and 0.49, respectively) (Fig. 3F).

### Collaborations

**FIGURE 4.**
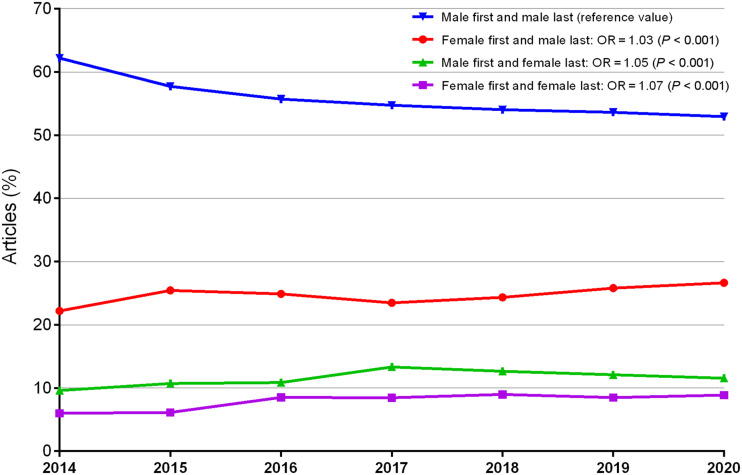
Percentage of collaboration between male and female first and last authors for articles published from 2014 to 2020. Male-to-male collaboration declined over time from 62.2% in 2014 to 52.9% in 2020 in favor of all other collaboration types. OR = odds ratio.

Assuming a linear evolution of outcomes over the study period and the year as a continuous factor, we observed a decrease in the proportion of articles produced by male first and last authors in favor of female first and last authors (odds ratio, 1.07; *P* < 0.001), male first and female last authors (odds ratio, 1.05; *P* < 0.001), and female first and male last authors (odds ratio, 1.03; *P* < 0.001). Indeed, 944 of 1,518 articles (62.2%) were produced by male first and last authors in 2014, and 1,094 of 2,069 (52.9%) in 2020. On the other hand, there was an increase in the proportion of articles produced by female first and male last authors from 337 of 1,518 articles (22.2%) in 2014 to 551 of 2,069 (26.6%) in 2020, by female first and last authors from 91 of 1,518 articles (6%) in 2014 to 184 of 2,069 (8%) in 2020, and by male first and female last authors from 146 of 1,518 articles (9.6%) in 2014 to 240 of 2,069 (11.6%) in 2020 (Fig. 4).

## DISCUSSION

There was a marked sex gap in first and particularly last authorship of articles published in nuclear medicine journals from 2014 to 2020. Women’s representation increased over time from 28.2% to 35.5% (*P* < 0.001) for first authors and from 15.6% to 20.5% for last authors, with a peak of 21.8% in 2017 (*P* < 0.001). Relative increases of 72% and 79% for female first and last authorship, respectively, were observed between 2014 and 2020. Parity was predicted in 2035 for first authors and in 2052 for last authors. A significant increase in female first and last authorship was observed in Europe and for publications in high-ranking journals. Female participation increased in original articles and case reports but not in reviews or editorials/letters. The proportion of articles produced by male first and last authors decreased by 10% in favor of all other collaborations.

To our knowledge, this was the first exhaustive bibliometric analysis of author sex in a wide spectrum of anglophone nuclear medicine journals over several years. Similar sex gaps in authorship have been reported in other domains of medicine and the STEM (science, technology, engineering, mathematics) sciences (4*,*^14^*,*^15^). For example, in 2018 Bendels et al. reported 33.1% female first and 18.1% female last authorship in high-quality research in 54 journals listed in the Nature Index in the categories “Life Science,” “Multidisciplinary,” “Earth and Environmental,” and “Chemistry” (4).

The lower percentage and rate of increase in female last authorship, a senior position, compared with female first authorship found in our study seem to confirm the presence of an invisible barrier for women to attain leadership positions: the so-called glass ceiling. Moreover, female last authorship increased from 2014 to 2017 but plateaued from 2017 to 2020. These findings could fuel the discussion recently launched by 3 European female nuclear medicine physicians about the challenges women currently face in this field dominated by men (16) and the steps that should be taken to allow female talent to achieve its full potential. Scientific societies, journal editors and publishing companies, scientific institutions, industry, funding agencies, and governments all have their role to play in the promotion of female scientific careers and the creation of a diverse and inclusive research environment. As an example, Gelardi et al. and Evangelista et al. have recently highlighted the underrepresentation of women on editorial boards of nuclear medicine journals, regardless of the rank within the board or the geographic provenance of the journal (17*,*^18^). Female participation varied from 14% to a maximum of 32%. Because our study shows that 1 in 3 first authors in nuclear medicine are female, female participation in all ranks of editorial boards should at least mirror this proportion.

Strategies could be put in place in all the aforementioned bodies to promote parity, such as providing transparency on women’s representation metrics, providing training on the benefits of diversity in health care, and even proposing sex quotas just as in politics. Obviously, those propositions are not miracle solutions for equality, but they are tools with potentially strong symbolic effects. It is worth to mention here some successes. The Athena SWAN Charter and Horizon Europe within the European Research Area are examples of initiatives aiming to overcome persisting sex gaps (19*,*^20^). Also, within nuclear medicine societies, several initiatives now exist such as the EANM Women’s Empowerment or the SNMMI Women in Nuclear Medicine, aiming to promote female networks and careers (21*,*^22^).

Female authors were equally represented among the journal ranks, and their participation increased for both high-ranking and other-ranking journals, suggesting that a possible sex bias during peer review did not result in an unbalanced representation of women across the journal ranks. However, although female participation increased for original articles and case reports, it did not change for reviews and editorials/letters. Furthermore, female first authors were underrepresented for editorials/letters. The productivity puzzle comprises many intricate pieces, and explanations for our findings are probably multifactorial. Sex differences in time management and publication patterns, thereby taking into account the cost–benefit ratio of different article types, may partly explain the unchanged female participation in reviews in favor of an increased female participation in original articles. Reviews are time-consuming to write but have less academic value than original articles in the same journal type. The potential gain in visibility by publishing a review might thus not be worth the investment when time is limited. When it comes to case reports, female participation was large and increased, although the academic value of this article type is low. Should we consider this a symptom of lower consideration by team leaders rather than a scientific achievement? Another explanation for our findings might be the invitation that can be required to write certain article types (8*,*^19^). A case-control study of sex disparities in invited commentaries showed that women had a 21% lower odds of receiving such an invitation than men despite having similar experience and that this disparity was greater for senior researchers (23).

An almost 10% decrease in the proportion of articles with a male first and last author was observed, in favor of all other collaborations. This finding might be due to the feminization of the workforce or an increased will of senior male team members to collaborate with female team members. Overall, the increasing tendency for collaboration with female first and last authors is encouraging.

This study had some principal limitations: the use of a software application to assign sex, the relatively short study period from 2014 to 2020 due to the absence of last author provenance on PubMed before 2014, insufficient data for 3 of the 6 continents, the absence of nuclear medicine publications outside the 15 journals analyzed, and the absence of professional or demographic data on the workforce worldwide, which prevented subgroup analyses or comparisons. Furthermore, there are no available data thus far on factors that impact career choices and evolutions in nuclear medicine. Nor are there any data on sex inequity in the nuclear medicine workforce, such as measures of unconscious bias, sexual/racial harassment, and the sex division of domestic labor impacting scientific productivity. National and international nuclear medicine associations could follow in the footsteps of the European Society for Medical Oncology by conducting a survey of male and female workers on sex-related challenges (24). Lastly, 2020 was marked by lockdowns due to the coronavirus disease 2019 pandemic. However, a preliminary analysis showed no alterations in the quantity of publications in medical imaging by female authors during this period (25).

## CONCLUSION

Although scientific production in nuclear medicine is no exception to sex inequity, the absolute numbers and proportions of female-authored publications substantially increased from 2014 to 2020, thereby narrowing the sex gap. Parity can be foreseen in a few decades.

## DISCLOSURE

Rachida Lebtahi is supported by Advanced Accelerator Applications, Ipsen, Sirtex, and Boston Scientific. Catherine Ansquer is supported by Advanced Accelerator Applications, Novartis, Ipsen, and Eisai. Elske Quak is supported by Advanced Accelerator Applications. No other potential conflict of interest relevant to this article was reported.

## Notes

KEY POINTS **QUESTION:** What are recent trends in the sex distribution of first and last authorship for articles published in nuclear medicine journals? **PERTINENT FINDINGS:** Although scientific production in nuclear medicine is no exception to sex inequity, our bibliometric study showed a substantial increase in female first and last authorship for articles published in nuclear medicine journals. We observed a wider sex gap for last than for first authorship. Although the sex gap in the authorship of original articles and case reports has narrowed over time, particularly in Europe, parity is still a few decades away. **IMPLICATIONS FOR PATIENT CARE:** Diversity, equity, and inclusion can drive innovation and improve the quality of care for our diverse patient population.
